# Renal Cells Express Different Forms of Vimentin: The Independent Expression Alteration of these Forms is Important in Cell Resistance to Osmotic Stress and Apoptosis

**DOI:** 10.1371/journal.pone.0068301

**Published:** 2013-07-11

**Authors:** Bettina S. Buchmaier, Asima Bibi, Gerhard A. Müller, Gry H. Dihazi, Marwa Eltoweissy, Jenny Kruegel, Hassan Dihazi

**Affiliations:** Department of Nephrology and Rheumatology, Georg-August University, Göttingen, Germany; Hertie Institute for Clinical Brain Research and German Center for Neurodegenerative Diseases, Germany

## Abstract

Osmotic stress has been shown to regulate cytoskeletal protein expression. It is generally known that vimentin is rapidly degraded during apoptosis by multiple caspases, resulting in diverse vimentin fragments. Despite the existence of the known apoptotic vimentin fragments, we demonstrated in our study the existence of different forms of vimentin VIM I, II, III, and IV with different molecular weights in various renal cell lines. Using a proteomics approach followed by western blot analyses and immunofluorescence staining, we proved the apoptosis-independent existence and differential regulation of different vimentin forms under varying conditions of osmolarity in renal cells. Similar impacts of osmotic stress were also observed on the expression of other cytoskeleton intermediate filament proteins; e.g., cytokeratin. Interestingly, 2D western blot analysis revealed that the forms of vimentin are regulated independently of each other under glucose and NaCl osmotic stress. Renal cells, adapted to high NaCl osmotic stress, express a high level of VIM IV (the form with the highest molecular weight), besides the three other forms, and exhibit higher resistance to apoptotic induction with TNF-α or staurosporin compared to the control. In contrast, renal cells that are adapted to high glucose concentration and express only the lower-molecular-weight forms VIM I and II, were more susceptible to apoptosis. Our data proved the existence of different vimentin forms, which play an important role in cell resistance to osmotic stress and are involved in cell protection against apoptosis.

## Introduction

Changes in osmolarity cause multiple alterations in cell metabolism and function, accompanied by changes in cell volume that are associated with rearrangement of the cytoskeleton [**1**]. Kidney is a key component of the defense system against osmotic stress conditions, due to its ability to produce urine of highly variable osmolarity, depending on the hydration status. The part of the nephron that plays a vital role in this remarkable feature of the kidney is the renal medulla [2]. The thick ascending limb of Henle’s loop (TALH) is a part of the outer renal medulla. It is involved in urinary concentration, mainly by reabsorption of ions and very poor water permeability of the luminal membrane. Thus, TALH cells are physiologically exposed to variable osmotic stress during diuresis or antidiuresis but also pathological circumstances; glucosuria in patients suffering from diabetes mellitus may occur. Cells undergoing fluctuations in osmolarity have developed several strategies for protecting themselves from the osmotic effect. One of them is the downregulation of the endoplasmic calcium-binding protein calreticulin [3]. Beside the sodium ion, chloride ion, and urea transport in the interstitium [4], the cells balance the osmotic stress by accumulation of organic osmolytes, such as sorbitol, betaine, inositol, glycerophosphocholine, and taurine [5]. These osmolytes are involved in counterbalancing regulatory volume decreases following hypertonic stress but are also released from cells undergoing regulatory volume increases caused by hypotonic stress [4]. Apart from modulation of the osmolyte content, cytoskeletal reorganization, such as a rearrangement of the F-actin cytoskeleton, occurs in renal medullary cells during osmoregulation [6], [7]. Using proteomics, we demonstrated in our previous studies that renal cells exhibiting high resistance to osmotic stress respond with alteration of the expression of cytoskeletal proteins, like vimentin (VIM) and cytokeratin (CK), to osmotic stress [8].

Cytoskeletal proteins build a dynamic filamentous network in the eukaryotic cell. They provide mechanical stability and the maintenance of the cell shape but are also involved in cell movement and transport mechanisms in the cytoplasm. In addition to mechanical features, the cytoskeleton also provides a surface for many signaling molecules, therefore controlling intracellular signaling events [9]. Intermediate filaments (IFs) belong to the major structural components of the cytoskeleton, along with microfilaments and microtubules. They are organized into complex arrays of 10-nm-diameter filaments that are prevalent in the perinuclear region, where they appear to be attached to the outer nuclear membrane, forming radial extensions through the cytoplasm [10]. Although IFs provide mechanical stability, there is evidence that they have dynamic properties, like the incorporation of newly synthesized subunits in pre-existing IF networks [11], [12]. Organizational changes in IF networks do not take place only during mitosis [13] and cell differentiation [14] but can also be provoked, for instance, by heat shock [15] or virus infection [16].

VIM is the major IF protein known to play role in many different aspects of cell physiology, cellular interactions, and organ homeostasis. VIM is a highly conserved protein with a very high degree of sequence homology between species, suggesting some important and evolutionary conserved physiological roles of this IF protein. VIM knockout mice studies revealed a key importance of the protein in several cellular functions due to morphological defects in glial cells, leading to damaged motor coordination, impaired ability to heal wounds, and changes in fibroblast migration capacity [17]. VIM also plays an important role in mechanical stability, migration, and motility of cells [17], [18]. Moreover, VIM is known as a classical landmark of epithelial-mesenchymal transitions (EMTs) and tumor progression [19].

However, the role of cytoskeletal proteins in the osmoadaptive response is poorly characterized. The purpose of the present study is to investigate the role of cytoskeletal proteins in the osmotic stress response and adaptation of renal cells, with emphasis on the modulation of VIM and its forms under different and variable osmotic stress conditions. We demonstrate that VIM is present in different forms that can be regulated independently from each other under osmotic stress conditions. Moreover, we highlight the role of the higher-molecular-weight VIM forms in cell resistance to apoptosis.

## Experimental Procedures

### Cell Line and Culture Procedure

Two renal epithelial cell lines–TALH (thick ascending limb of Henle’s loop), derived from rabbit kidney’s outer medulla, and HK2 (human kidney-2), derived from normal adult human renal cortex–were used in these experiments. An immortalized TALH cell line, derived from kidneys of New Zealand white rabbits, was used [20]. The HK2 (human kidney 2) cell line was purchased from American Type Culture Collection (ATCC, Manassas, Virginia USA) [21]. The renal fibroblasts cell line TK173, derived from human kidney, was also used in these experiments. TK173 cells were immortalized by transfection with the plasmid pSV3gpt from SV 40 and have typical morphological and biochemical properties of renal interstitial fibroblasts [22]. All three cell lines show a high degree of differentiation and specialization and provide a suitable tool to study renal function in vitro. TALH and TK173 cell lines were maintained as a monolayer culture in DMEM (Gibco), including 5.5 mmol/l D-glucose supplemented with 10% fetal calf serum (Roche), 1% MEM nonessential amino acids, and 1% L-glutamine, whereas the HK2 cell line was maintained as a monolayer culture in Quantum 286 for Epithelial Cells medium (PAA). All cultured media were provided with 1% penicillin/streptomycin (Gibco). Cells were passaged at 85% to 90% confluency. Cells were routinely cultured in 75 cm^2^ tissue culture flasks (Falcon) at 37°C in a humidified 5% CO_2_/95% air atmosphere.

### Osmotic Stress Experiments

After reaching 70% confluence, cells that were cultivated in 300 mosmol/kg medium (TALH-STD, TK173-STD and HK2-STD) were stressed with either 600 mosmol/kg (NaCl or urea) or 30 mM glucose medium. Cell lines exhibiting high resistance to osmolarity (600 mosmol/kg) (TALH-NaCl, TALH-Urea, TK173-NaCl and HK2-NaCl) were established. Furthermore, the TALH cell line, adapted to high glucose 30 mM (TALH-Glucose), was established. For hypoosmolarity experiments, cell lines that were adapted to high osmolarity were switched back to 300 mosmol/kg medium. The osmolarity was adjusted with 3 M NaCl, 6 M urea, or D-glucose and controlled routinely.

### Protein Extraction

Seventy percent-confluent cultures were scraped and washed 3 times with PBS. The cells were harvested by centrifugation at 200×g for 10 min, and the pellet was treated with 0.3–0.5 ml lysis buffer containing 9.5 M urea, 2% (w/v) CHAPS, 2% (v/v) ampholytes, 1% (w/v) DTT, and 10 mM PMSF. After adding the lysis buffer, the samples were incubated for 30 min at 4°C. For removing the cell debris, sample centrifugation was carried out at 13,000×g and 4°C for 30 min. The supernatant was recentrifuged at 13,000×g and 4°C for an additional 30 min to get maximal purity. The resulting samples were either used immediately or stored at −80°C until use. Protein concentration was measured according to Bradford [23] using bovine serum albumin as a standard.

### Two-dimensional Gel Electrophoresis and Mass Spectrometry Analysis

For 2-DE, protein extracts were diluted in rehydration buffer (8 M urea, 1% (w/v) CHAPS, 0,2% ampholytes pH 3–10 or pH 5–8 according to the IPG strips, 15 mM DTT, and a trace of bromophenol blue) to a final volume of 175 µl for 11-cm strips and 125 µl for 7-cm strips. The mixture, containing 150 µg protein for 11-cm strips (pH 5–8) or 40 µg protein for 7-cm strips (pH 3–10), was used for the rehydration of the immobilized pH gradient (IPG) strips (Bio-Rad). The strips were allowed to rehydrate for 1 h before adding mineral oil. The passive rehydration was carried out overnight for at least 12 h at room temperature in a focusing chamber. Isoelectric focusing with a Protean IEF was performed at 20°C using the following multistep protocol for 11-cm strips: 500 V for 1 h, 1000 V for 1 h, and 8000 V for 6 h. The following protocol was used for 7-cm strips: 200 V for 1 h, 500 V for 1 h, 1000 V for 1 h, and 4000 V for 3 h. After the first dimension, the individual strips were equilibrated in 6 M urea, 30% (w/v) glycerol, 2% (w/v) SDS, 0.05 M Tris-HCl pH 8.8, and 15 mM DTT for 20 min. An additional incubation in the same buffer, supplemented with iodoacetamide (40 mg/ml), was carried out for another 20 min. After equilibration, the IPG strips were loaded on 12% SDS-PAGE, which was performed for 1 h at 200 V and 4°C.

### Gel Staining and Analysis

For image analysis, 2-D gels were fixed in a solution containing 50% methanol and 12% acetic acid overnight and fluorescently stained with Flamingo fluorescent gel stain (Bio-Rad, Hercules, CA, USA) for a minimum of 5 h. After the staining, gels were scanned at 50-µm resolution on a Fuji FLA5100 scanner. The digitalized images were analyzed; spot matching across gels and normalization were performed using Delta2D 3.4 (Decodon, Braunschweig, Germany). Delta2D computes a ‘spot quality’ value for every detected spot. This value shows how closely a spot represents the ‘ideal’ 3D Gaussian bell shape. Based on average spot volume ratio, spots whose relative expression is changed at least 2-fold (increase or decrease) between the compared samples were considered to be significant.To analyze the significance of protein regulation, Student’s t-test was performed, and statistical significance was assumed for P values less than 0.01. 2-D gels were post-stained with colloidal Coomassie blue (Roti-Blue, Roth, Karlsruhe, Germany) overnight, and differentially regulated proteins were excised and processed for identification by MS.

### In-gel Digestion and Mass Spectrometry Analysis of Protein Spots

In-gel digestion experiments were performed according to Dihazi et al., 2005 [8]. Briefly, Coomassie brilliant blue-stained spots were manually excised from the gels and washed with distilled water for 15 min. The destaining procedure was carried out by washing the spots alternately with 50% ACN (acetonitrile) and 100 mM ammonium bicarbonate 3 times for 5 min. After dehydrating the spots with ACN for 15 min, they were dried in a vacuum centrifuge for approximately 15 min. Thereafter, the gel spots were rehydrated for digestion with 40 µl trypsin (10 ng/µl in 100 mM ammonium bicarbonate) and incubated at 37°C overnight. The peptide samples were extracted with different concentrations of ACN and trifluoroacetic acid (TFA). The peptide samples were cocrystallized with the matrix (2, 5 diaminobenzoic acid) on a stainless steel target using 1 µl matrix and 1 µl sample. An Applied Biosystems Voyager-DE STR time-of-flight mass spectrometer, operating in delayed reflector mode with an accelerated voltage of 20 kV, was used to generate peptide mass fingerprint maps. Mass spectra were obtained by averaging 50 individual laser shots. All samples were externally calibrated with a peptide mix of des-Arg^1^-bradykinin ([M+H]+904.46), angiotensin I ([M+H]^+^1296.68), Glu^1^-fibrinopeptide B ([M+H]^+^1570.67), ACTH (1–17) ([M+H]^+^2093.08), and ACTH (18–39) ([M+H]^+^2465.19), and the resulting mass spectra were internally calibrated with trypsin autolysis products (m/z 842.50 and m/z 2211.10). Monoisotopic peptide masses were assigned, and database searches in the Swiss-Prot primary sequence database, restricted to the taxonomy Homo sapiens, were performed using MASCOT Software 2.2 (Matrix Science). Carboxamidomethylation of Cys and oxidation of Met were specified as variable modifications. One missed trypsin cleavage was allowed. Mass tolerance was set to 50 ppm for PMF searches. The minimal requirement for accepting a protein as identified was at least 30% sequence coverage in the PMF. Alternatively, tryptic peptides were subjected to mass spectrometric sequencing using a Q-TOF Ultima Global mass spectrometer (Micromass, Manchester, UK), equipped with a nanoflow ESI Z-spray. For that purpose, gel plugs were excised from 2-D gels and digested as described previously [21]. After digestion, the supernatant was removed and saved, and the additional peptides were extracted with increasing acetonitrile/trifluoroacetic acid solutions under sonication. All supernatants were pooled together, dried in a vacuum centrifuge, and re-dissolved in 0.1% formic acid for injection in the Q-TOF. The mass spectrometric sequencing was performed as described previously [20]. Processed data were searched against the MSDB and Swissprot databases through the Mascot search engine using a peptide mass tolerance and fragment tolerance of 0.5 Da. Protein identifications with at least two peptides sequenced were considered significant.

### Western Blot Analysis

Western blot analysis was performed according to Towbin et al. [24]. Forty micrograms of the cell extracts was loaded on an SDS gel after denaturation with Laemmli buffer. After SDS-PAGE, blotting was performed on nitrocellulose membranes (Amersham Pharmacia Biotech, Buckinghamshire, UK) at 40 V for 24 h in transfer buffer (25 mM Tris–HCl pH 8.4, 192 mM glycine, 0.5% SDS, 20% methanol). The membranes were blocked in 5% non-fat dry milk in PBS buffer containing 0.1% Tween-20 for 2 h at 37°C. The incubation with the primary antibodies was carried out overnight at 4°C. Monoclonal mouse anti-VIM (clone V9 for immunofluorescence, clone Vim 3B4 for WB) and monoclonal mouse anti-CK (clone MNF116) antibodies were from Dako. Monoclonal mouse anti-β-actin (product number A2228) antibody and monoclonal mouse anti-cofilin (CFL) (product number C8736) antibodies were from Sigma. Rabbit anti-lamin A/C polyclonal antibody was purchased from Cell Signaling Technology. After washing, membranes were incubated with a 1∶2000 dilution of horseradish peroxidase-conjugated sheep anti-mouse secondary antibody (Amersham Biosciences) or with a 1∶10,000 dilution of horseradish peroxidase-conjugated sheep anti-rabbit antibody for 60 min at 37°C. To visualize the protein bands, nitrocellulose membranes were then washed and treated with western blotting luminal reagent (Perkin Elmer, Boston, USA). Finally, the results were obtained on Kodak films.

### Indirect Immunofluorescence Staining

For the indirect immunofluorescence staining, 30,000 cells were cultivated overnight in 16-well chamber slides. The medium was removed, and the cells were washed twice with PBS buffer. Fixation of the cells was carried out for 15 min with 4% paraformaldehyde in PBS. After permeabilization with 0.1% Triton X-100 in PBS for 1 h, the fixed cells were blocked with 1% BSA/PBS-buffer for 30 min and incubated overnight separately with the primary antibody. After washing 3 times with PBS, cells were incubated for 1 h with Alexa fluor dye-coupled anti-mouse and anti-rabbit secondary antibodies (Invitrogen, Germany). The unbound secondary antibody was removed with 3 washes of PBS buffer for 10 min. Thereafter, the samples were counterstained with PPD mounting medium containing DAPI. Samples were analyzed on an inverted immunofluorescence Zeiss Axiophot microscope (Carl Zeiss, Jena, Germany) equipped for epifluorescence with objectives ranging from magnifications of 10× to 100× with oil-immersion and a black and white Zeiss Axiocam CCD camera. Image capture was carried out using AnalySIS software (Soft Imaging Systems, Leinfelden, Germany).

### siRNA Construct and Transfection

siRNA oligonucleotides specific for knocking down VIM expression were designed in our laboratory and synthesized by Eurofins MWG Operon (Germany). We designed three different oligonucleotides, which target different parts of the vimentin mRNA sequence:

siRNA1: 5′-TGAAGCTGCTAATTACCAAGACACTAT-3′ siRNA2: 5′-TCCAGGAACAGCATGTCCAGATCGACG-3′ siRNA3: 5′-ATCAACACCGAGTTCAGGAACACCCGC-3′. TALH-STD cells cultured to approximately 80% confluency were transfected with siRNA for VIM knockdown using transfection reagent Lipofectamine 2000™ (Invitrogen) according to the standard protocol of the manufacturer. Cells treated with Lipofectamine alone without siRNA were used as positive control. The transfection medium was removed after 24 h and replaced with the appropriate medium (control, glucose or NaCl). Cells were maintained under osomotic stress or in control medium for additional 24 h. The VIM expression was assessed by Western blot. The impact of VIM knockdown on cell viability and proliferation under osmotic stress was investigated in 96 well pate using MTT cell viability assay.

### MTT Cell Viability Assay

For the cell viability assay the cell proliferation Kit I (MTT) from Roche was used according to the manufacturer instructions. To investigate the effect of knocking down vimentin on cell viability under osmotic stress conditions, 8000–10.000 cells were grown in a 96 well tissue culture plate in control medium. After 24 siRNA treatment in optimum medium, the cells were switched back either to control medium or to high osmotic medium (glucose or NaCl). After 24 h osmotic stress the MTT test was performed according to the manufacturer recommendation. All experiments were repeated three times and during each experiment 5 replicate per case were carried out.

### Apoptosis Assay

To investigate the role of VIM in cell resistance to apoptosis TALH cell maintained in culture plate in control medium were divided in 4 groups: the control group without any treatment, NaCl-group treated for 24 h with sodium chloride, siRNA-group treated for 24 with siRNA and siRNA-NaCl group treated first with siRNA for 24 h and then subjected to NaCl stress for additional 24 h. The cells from the different groups were harvested, protein extracts were prepared and Western blot for caspase 3, 8 and 9 was performed using the following antibodies: anit-capase 3 (product number 9662) and anti-caspase 9 (product number 9508) monoclonal antibodies (Cell Signaling, USA) and anti-caspase 8 antibody (product number sc-7890) (Santa Cruz, USA).

### Apoptosis Induction and Caspase and Protease Inhibition in TALH Cells

For apoptosis induction, TALH cells were treated with either 100 ng/ml of tumor necrosis factor-α (TNF-α) for the extrinsic pathway or 1 µM staurosporine for the intrinsic (mitochondrial) pathway for 0, 2, 4, 6, or 8 h.

The caspase inhibition was achieved by treating the cells for 2 h with Z-Val-Ala-Asp (OMe)-FMK (Z-VAD-fmk) (Enzo Life Sciences) and then for an additional 4 h with either 100 ng/ml TNF-α together with 10 µg/ml cycloheximide or 1 µM staurosporine.

For protease inhibition experiments, cells were treated for 4 h with 100 µM N-Tosyl-L-Phenylalanine Chloromethyl Ketone (TPCK) or N-a-Tosyl-L-Lysine Chloromethyl Ketone Hydrochloride (TLCK). Cell viability was determined by an MTT assay kit according to the manufacturer’s recommendations. All experiments were repeated 3 times.

### Statistical Analysis

All blots were quantified using the ImageJ software. Graphpad prism was used for graphical presentation and analysis by Student’s t-distribution. Results are expressed as the average (mean±SD) of three or more independent experiments. Differences were considered statistically significant when p<0.05.

## Results

### Differential Expression Regulation of Cytoskeletal Proteins under Different Osmotic Stress Conditions

**Figure 1:**
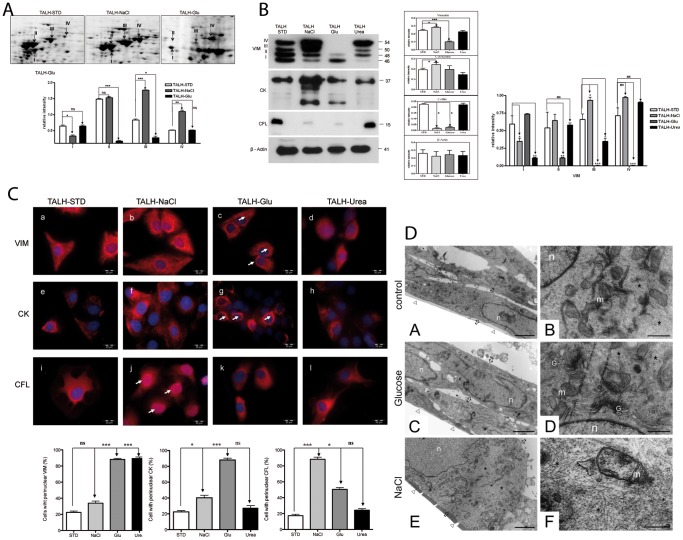
Differential expression regulation of cytoskeletal proteins under different osmotic stress conditions. A: Enlargments of gel regions in the range of pH 5–8 that were cropped from Flamingo fluorescence dye stained 2D-gels. The panels show differential expression of VIM forms in control cells (TALH-STD) and cells cultured in hyperosmolarity medium of NaCl (TALH-NaCl) and glucose (TALH-Glu). Quantitative analyses were carried out by comparing the VIM expression changes between TALH-STD and –NaCl and between TALH-STD and –Glu. The expression quantification of the spots is presented as a grouped bar chart with error bars. Each bar represents the intensity means ± S.D. of vimentin protein spots from 3 independent experiments. Significant differences: (*) p<0.05, (**) p<0.01, (***) p<0.001. B: Ci Western blot analysis of cytoskeletal proteins VIM, CK, and CFL in TALH-STD, TALH-NaCl, TALH-Glu, and TALH-Urea cells. VIM showed 4 different bands, called forms I (46 kDa), II (48 kDa), III (50 kDa), and IV (54 kDa), from lower to higher molecular weight, respectively. VIM forms showed differential regulation under different stress conditions, where β-actin, used as control, was equally expressed. Cii the WB bands from the different VIM forms (I–IV) were quantified separately. CK showed four different bands in Western blot, all the four bands were up-regulated in TALH-NaCl. In TALH-Glu cells only the form with lowest molecular weight showed slightly up-regulation, when compared to TALH-STD. CFL expression level was significantly down-regulated in TALH-NaCl –Glu. The expression quantification is presented as a grouped bar chart with error bars on the left. Each bar represents the intensity means ± S.D. of blots from 3 independent experiments. Significant differences: (*) p<0.05, (**) p<0.01, (***) p<0.001. C: Immunofluorescence staining of TALH-cells using mouse anti-VIM (a, b, c, d), anti-CK (e, f, g, h), and anti- CFL (i, j, k, l) antibodies in TALH-STD, TALH-NaCl, TALH-Glucose, and TALH-Urea cells, respectively. VIM builds a strong filamentous network in TALH-NaCl (b) cells compared to strong perinuclear restriction in TALH-Glu (c) cells. The number of cells with perinuclear localisation of the stained protein (VIM, CK or CFL) was counted and presented in per cent of the total number of cells in the wells. The stressed cells were compared with the TALH-STD. Each bar represents the number of cells with perinuclear localization in % (± S.D of 200 counted cells/well from 3 independent wells. Significant differences: (*) p<0.05, (**) p<0.01, (***) p<0.001. Arrows indicate the cell with perinuclear localisation of IFs. In case of (J) the arrows indicate the perinuclear localisation of CFL. All the scale bars represent 20 µm; D: TALH-STD, -NaCl- and -Glu cells, respectively, were seeded on 96-well plates and allowed to attach for 24 h. The cells were processed as described in material and methods. Slices were contrasted with uranyl acetat and lead citrate. Analysis of the section was done with a LEO906 E electron microscope. Right and left panel represents same cells with different magnification n: nucleus, m: mitochondria G: golgi apparatus, Δ: plastic foil (where the cells attach), arrow: indicate the border of the cells *: indicate the IFs localization. All the scale bars on the left panel represent 2 µm and on the right panel represent 5 µm.

To investigate the changes in the protein profile upon osmotic stress, we performed the proteomic characterization of TALH cells exposed for long term to hyperosmotic glucose (TALH-Glu) and NaCl (TALH-NaCl) stress and compared them to the control (TALH-STD). Cell extracts were prepared from TALH-STD, TALH-Glu, and TALH-NaCl cells and separated by 2D gel electrophoresis. The examination of the gels in the pH range of 5–8 revealed significant differences between the protein profiles of stressed cells compared to non-stressed cells. Using mass spectrometry analysis and database searches, we identified up to 4 differentially regulated spots as one of the major intermediate filament proteins, VIM (Fig. 1A, Fig. S1 and Table 1). Moreover, quantification of regulated VIM spots showed that they are regulated independently of each other. 2D gel analysis further revealed that VIM forms (VIM I–IV) were differentially regulated under glucose and NaCl stress.

**Table 1:** List of differentially expressed proteins identified in TALH-Glu cell line compared to TALH-STD cells (pH range 5–8).

Spot	Protein name	Molecular mass	Up/Down regulation	Score fingerprint	Accession no
				(p<0,05)	NCBI
1	78 kDa glucose related protein precursor (GRP78)	72505	↓	180	gi 121570
2	Heat shock protein 8	71055	↓	154	gi 13242237
3	Stress-70 protein, mitochondrial precursor	73768	↓	101	gi 14917005
4	78 kDa glucose related protein precursor (GRP78)	72455	↓	80	gi 115502217
5	60 kDa heat shock protein, mitochondrial precursor	61122	↓	108	gi 129378
6	Vimentin	44611	↓	125	gi 860908
7	Vimentin	44611	↓	239	gi 860908
8	ATP synthase beta subunit	51171	↓	92	gi 1374715
9	Keratin complex 1, acidic, gene 19, isoform CRA_c	25547	↓	73	gi 149054202
10	Prohibitin	29859	↓	153	gi 6679299
11	EndoA’ cytokeratin (5′ end put.); putative	53210	↓	114	gi 53210
12	Alpha-enolase	47449	↓	108	gi 162949733
13	Alpha-enolase	47449	↓	108	gi 162949733
14	2010209012Rik protein	62081	↓	68	gi 18043971
15	Cofilin	1856	↓	79	gi 291415601
16	THUMP domain containing 2	42428	↑	77	gi 58865786
17	Keratin 8	54514	↑	120	gi 76779293
18	Unnamed protein product	32580	↑	68	gi 26355849
19	Protein disulfide-isomerase precursor (PDI)	57374	↑	78	gi 62287156
20	Gamma actin, cytoplasmic 1	32941	↑	77	gi 123298587
21	Proteasome (prosome, macropain) subunit, alpha type 6	27838	↑	75	gi 123298587

Western blot analysis was performed to confirm the results obtained with the 2D gels. The obtained data showed a differential regulation of different cytoskeletal proteins, such as VIM, CK, and CFL, under different types of osmotic stresses. VIM was expressed as four different forms (termed VIM I–IV), with VIM I representing the form with lowest MW (46 kDa) and VIM IV the form with the highest MW (54 kDa). Under osmotic stress the expression oft the VIM forms seemed to be regulated differentially and independently from each other. VIM I was significantly down-regulated in TALH-NaCl and TALH-Urea cells but not in TALH-Glu cells. VIM II was down-regulated in TALH-Glu cells. Whereas in comparison to control cells VIM III was upregulated in TALH-NaCL cells, it was downregulated in TALH-Glu and TALH-Urea cells. In case of VIM IV, expression levels were only found to be changed in case of TALH-Glu cells. Similar to VIM, CK showed four different bands in Western blot, all the four bands were up-regulated in TALH-NaCl, whereas in TALH-Glu cells only the form with lowest molecular weight showed slightly up-regulation. In TALH-Urea cells the expression of CK was not significantly altered. However, cofilin (CFL) was down-regulated in both TALH-NaCl and TALH-Glu cells but not in TALH-Urea cells (Fig. 1B).

Expression and intracellular localization of cytoskeletal proteins under different osmotic stresses were also examined by indirect immunofluorescence staining. VIM and CK were localized in the cytoplasm, with perinuclear staining in TALH-Glu cells (Fig. 1C c, g, Fig. S2 A,B) compared to an evenly distributed cytoplasmic network in TALH-STD (Fig. 1C a, e, Fig. S2 A,B) and particularly in TALH-NaCl (Fig. 1C b, f, Fig. S2 A,B) cells. CFL showed a predominantly nuclear localization in TALH-NaCl cells (Fig. 1C j), while the other TALH cell lines showed homogenous staining of the cytoplasm (Fig. 1C i, k, l). Electron microscopic analysis of the TALH cells under different stress conditions confirmed stress dependent differences in the density and distribution of intermediate filaments. Electron micrographs showed that, compared to TALH-STD and –Glu cells, the cytoplasm of TALH-NaCl is filled with intermediate filaments (Fig. 1D).

### Time-dependent Expression Alteration of VIM Forms Under Osmotic Stress Conditions

**Figure 2:**
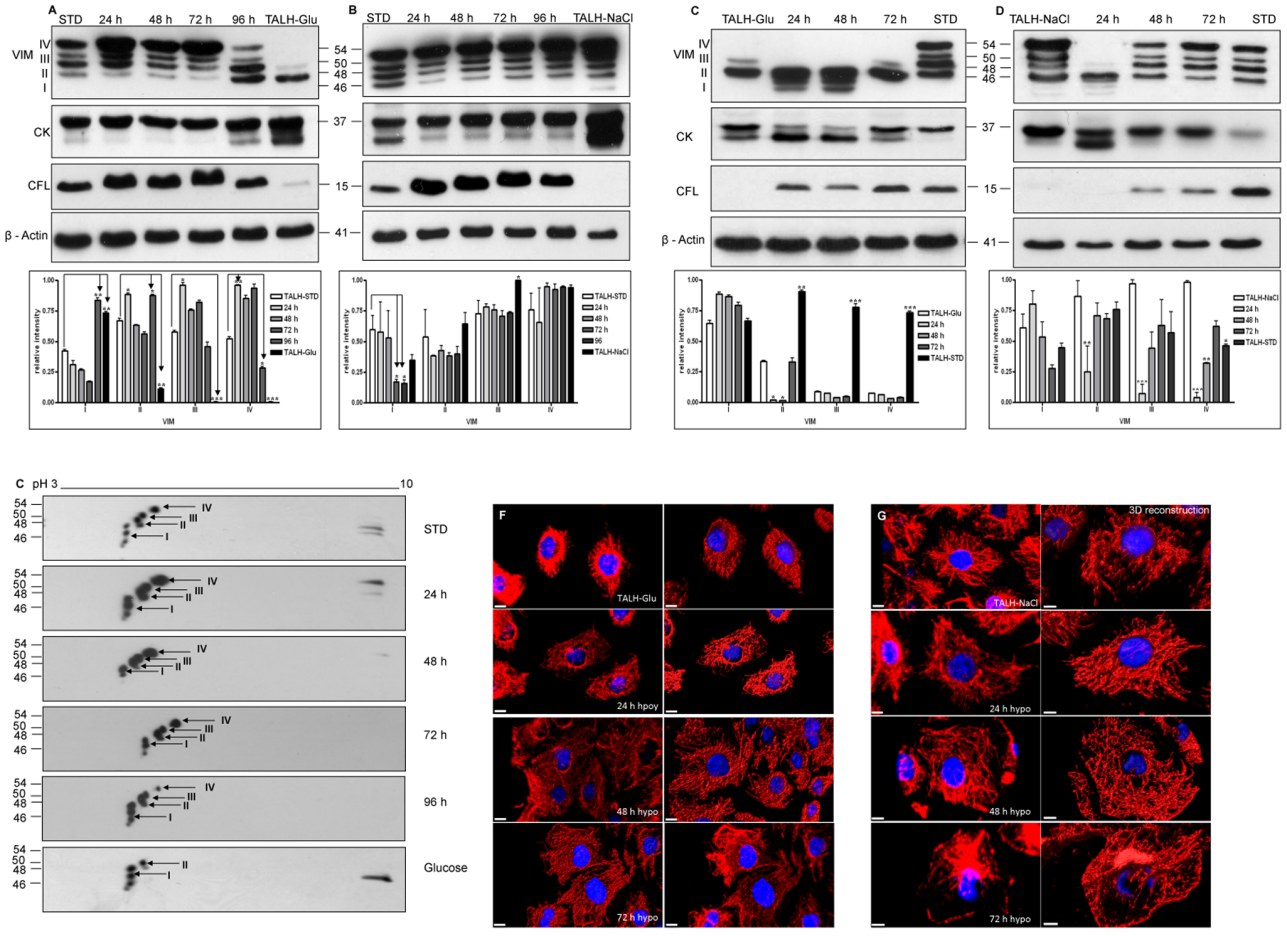
Differential expression of VIM forms under time-dependent osmotic stress conditions. Western blot analysis of cytoskeletal proteins during osmotic stress adaptation. A: TALH-STD cells were stressed with 30 mM glucose medium and tested for protein expression changes after 0, 24, 48, 72, and 96 h with antibodies against the indicated protein. B: TALH-STD cells were stressed with 600 mosmol/kg NaCl medium and analyzed after 0, 24, 48, 72, and 96 h. C: Adapted TALH-Glu cells were switched back to hypoosmolar medium with 300 mosmol/kg medium and tested for protein expression changes after 0, 24, 48, and 72 h. D: Adapted TALH-NaCl cells were switched back to hypoosmolar (300 mosmol/kg) medium and tested for protein expression changes after 0, 24, 48, and 72 h. E: 2D western blot analysis of VIM expression in TALH-STD cells in the course of hyperosmolar glucose stress. TALH-STD cells were stressed with 30 mM glucose medium and tested for VIM expression after 0, 24, 48, 72, and 96 h. VIM IV subtype (arrowhead) increases after 24 h during hyperosmolar glucose stress and then decreases to almost absent in TALH-Glu cells that are cultured more than 96 h in glucose media. Immunofluorescence staining of VIM in TALH cells. F: cells switched from Glucose to hypoosmolar medium for 24, 48, and 72 h, the scale bars represent 10 µm. G: cells switched form 600 mosmol/kg NaCl to hypoosmolar medium for 24, 48, and 72 h, the scale bars represent 5 µm. Immunofluorescence staining of cells was then carried out using anti-VIM antibody. Image analysis was carried out as described in material and method part.

In order to follow the expression regulation of cytoskeletal proteins under osmotic stress, TALH-STD cells were cultured for different time periods under glucose or NaCl osmotic stress. Western blot analysis of samples collected after 24, 48, 72, and 96 h of osmotic stress showed that the different VIM forms were regulated in a time-dependent manner. The expression of VIM III and VIM IV, which are almost not expressed in TALH-Glu cells, was up-regulated in the first 72 h, followed by a sustained down-regulation after 96 h of glucose stress. On the other hand, VIM form II was transiently up-regulated after 24 h of glucose stress and VIM I was down-regulated during the first 72 h of stress, both forms were followed by a significant upregulation after 96 h (Fig. 2A). Whereas the cells cultured under NaCl stress conditions showed transient down-regulation of the VIM forms I and II (Fig. 2B).

When higher osmotic stress adapted TALH cells (TALH-Glu or TALH-NaCl) were cultured back to hypoosmotic standard medium, lower-molecular-weight forms of VIM increase transiently after 24 h in TALH-Glu cells (Fig. 2C) in contrast to rapid and transient down-regulation of upper forms of VIM (II, III and IV) in TALH-NaCl cells (Fig. 2D).

In parallel to VIM, the expression of 2 other cytoskeletal proteins, CK and CFL, was also monitored during the course of hyperosmolar treatment of TALH-STD cells with either 600 mosmol/kg NaCl medium or 30 mM glucose medium and back to hypoosmolar medium. We observed differential regulation of different forms of CK under both types of hyperosmotic stress (Fig. 2A, 2B). CFL was also found to be up-regulated, along with a shift in the molecular weight (Fig. 2A, 2B). In the course of hypoosmolar stress, however, CK showed transiently a lower MW band, whereas CFL down-regulation was recovered after 24 h in TALH-Glu cells (Fig. 2C) and 48 h in TALH-NaCl cells (Fig. 2D).

2D gel electrophoresis and western blot analyses demonstrated the presence and regulation of different forms of VIM under hyperosmotic stress conditions with glucose or NaCl. To further validate these data, 2-D western blot analysis was performed. The obtained data confirmed the presence of different forms of VIM and their independent regulation. The subtype with higher molecular weight (VIM IV) was first up-regulated under glucose stress and disappeared when the cells were cultured for more than 96 h under these conditions (Fig. 2E). Furthermore, the expression of the basic forms of VIM in the pH range of 9 was also found to be altered under glucose stress (Fig. 2E), whereas the expression of acidic forms was altered in the course of NaCl stress (Fig. S3 A).

To monitor the alterations of VIM distribution in TALH cells, immunofluorescence analysis was performed during hypoosmolar stress of TALH-Glu and TALH-NaCl cells (Fig. 2F, 2G). Glucose or NaCl adapted cells were stained by immunofluorescence with anti-VIM antibody after 0, 24, 48, and 72 h of hypoosmolar stress caused by 300 mosmol/kg medium. Cells that were adapted to higher glucose concentrations (TALH-Glu) showed a compact network of VIM filaments in the perinuclear region. Twenty-four hours after shifting the cells to hypoosmolar stress conditions, VIM takes filamental structure and spreads in entire cell. After 48 h, VIM filaments started to redistribute into a uniform network, which achieved an optimal distribution after 72 h. Cells did not build the compact network around the nucleus again, but they showed a TALH-STD-like phenotype (Fig. 2F). Cells adapted to high NaCl concentration swell significantly after 48 h in hypoosmotic medium as a consequence of water uptake. Parallel to cell swelling the vimentin filaments spread throughout the cytoplasmic periphery (Fig. 2G). Whereas after 72 h of hypoosmotic stress VIM was found on one hand to colocalise with plasma membrane and the other hand to build dense filaments in the periphery of the nucleus (Fig. 2G, Figure S3 B).

### The Impact of the Osmotic Stress on VIM is not Restricted to TALH Cells

**Figure 3:**
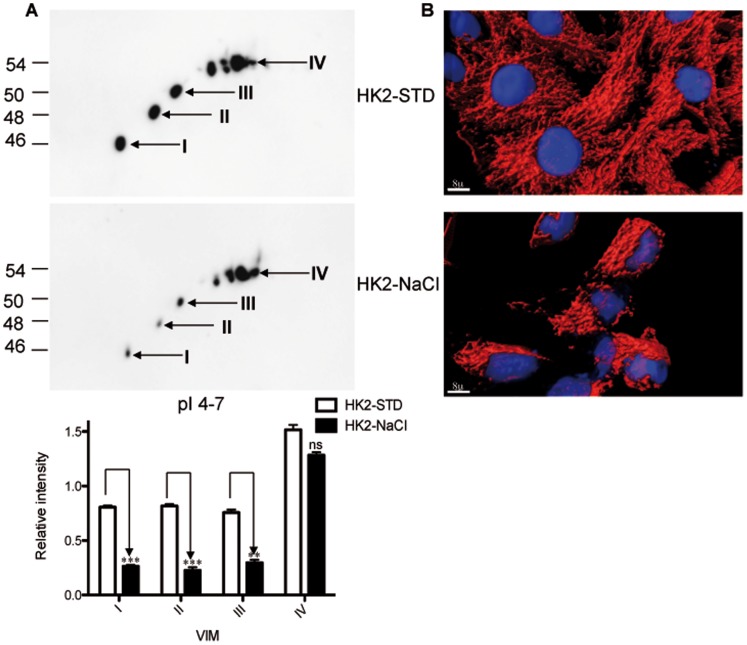
Differential expression of VIM forms under osmotic stress conditions in HK2renal epithelial cells. A: 2D western blot analysis of VIM expression in HK2-STD and HK2-NaCl cells in the pI range 4–7. Lower forms of VIM showed a decrease in expression in HK2-cells adapted to high NaCl. B: Immunofluorescence staining of VIM in HK2-NaCl cells showed an extended network compared to HK2-STD cells the scale bars represent 8 µm.

To investigate whether the VIM behaviour is specific for TALH cells, the impact of osmotic stress on the expression of VIM in HK2 cells from the renal proximal tubule was investigated. 2D western blot analysis of HK2 cells that were adapted to high NaCl stress medium also showed a down-regulation of lower VIM forms (Fig. 3A). Similar to TALH cells, the distribution of VIM in HK2 cells was affected by NaCl stress (Fig. 3B). HK2-NaCl cells showed a perinuclear distribution of VIM compared to a uniform cytosolic organization of the VIM filaments in HK2-STD cells.

**Figure 4:**
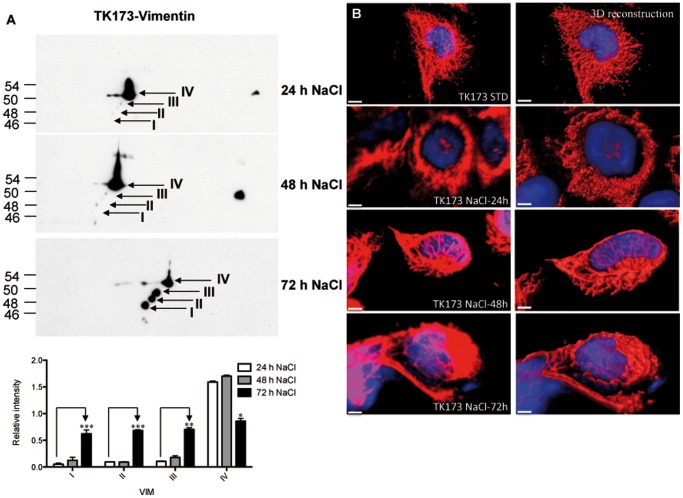
Differential expression of VIM forms under osmotic stress conditions in TK173 renal fibroblast cells. A: 2D western blot analysis of VIM expression in TK173 cells during hyperosmolar NaCl stress. After 72 h of NaCl stress, 4 VIM forms (numbered I–IV) appeared. B: Immunofluorescence staining of TK173 cells during NaCl stress. After 48 h, VIM builds a strong filamentous network around the nucleus, shown with a green arrowhead. The scale bars represent 3 µm.

In contrast to the epithelial cells (TALH and HK2), Western blot analyses of protein extracts from the fibroblast cell line TK173 showed an up-regulation of the lower VIM forms after 72 h of hyperosmolar NaCl stress (Fig. 4A). Immunofluorescence-stained TK173 cells showed a perinuclear reorganisation of VIM filaments after 24 h of hyperosmotic stress, this became intense with increased incubation time (Fig. 4B). TK173-NaCl cells showed filamentous and uniform VIM redistribution after 24 h when removed back to hypoosmolar medium (data not shown).

### VIM Forms Especially VIM III and IV Seems to be Important in Cell Survival and Adaptation Under Osmotic Stress

**Figure 5:**
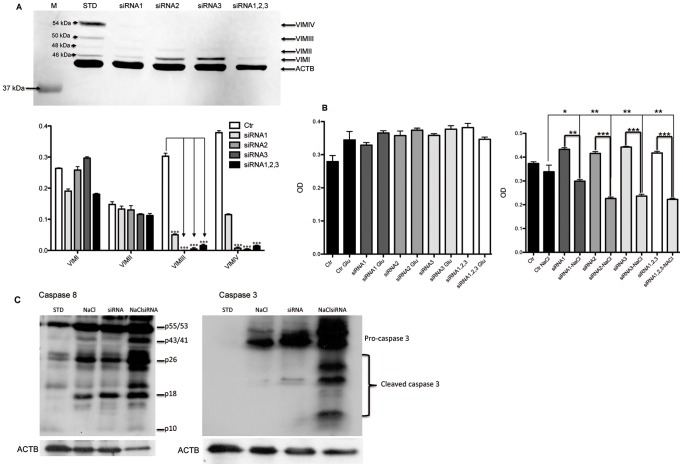
VIM knock-down using siRNA. A: Western blot analysis of VIM in non-transfected TALH-STD and TALH-STD cells transfected with the VIM siRNAs 1, 2, 3 or all three combined. B: siRNA transfected TALH-STD-cells and TALH-STD were subjected to either to NaCl or glucose stress. Cell viability and proliferation, determined with MTT assay, showed a slight increase in cell proliferation upon siRNA treatment but the transfected cells showed higher sensitivity to osmotic stress compared to control. C: The monitoring of apoptosis in siRNA TALH-STD-cells subjected to osmotic stress was carried out using Western blot for caspase 8 (left panel) and 3 (right panel). Each bar represents means ± S.D. of results from 3 independent experiments. Significant differences: (*) p<0.05, (**) p<0.01, (***) p<0.001.

In order to gain more insights in the role of VIM forms in cell adaptation and viability under stress conditions, siRNA knockdown of VIM followed by osmotic stress with NaCl and glucose were carried out. The oligonucleotides were designed to target different part of the VIM (begin siRNA3, middle siRNA2 and end siRNA1 of the mRNA) (Fig. S4 A). Experiments with single nucleotide and combined nucleotide were performed. The Western blot results showed over 95% know-down of VIM III and IV with all three designed siRNAs, whereas for VIM I and II no significant down-regulation could be achieved (Fig. 5A, Fig. S4 B). The cells with down-regulated VIM III and IV react differently dependent on the type of osmotic stress agent. High glucose concentrations did not affect the cell viability and proliferation, whereas high NaCl concentration seams to impact the cell viability and proliferation (Fig. 5B). Moreover the caspase activity assay confirmed an activation of caspase 8 and 3 in TALH-cells treated with siRNA and subjected to osmotic stress (Fig. 5C, Fig. S4 C). The caspase activation was evidenced by the appearance of the cleaved caspase 3 and 8. Western blot analysis did not show any activation of caspase 9 in siRNA treated cells under osmotic stress (data not shown). The data from siRNA, cell viability assay, and caspase activity assay suggest the role of VIM III and IV in the osmotic stress adaptation especially to NaCl. Down-regulation of VIM III and IV correlated with an increase in cell death under NaCl stress (Fig. 5 B). Furthermore the fact that all three designed siRNA did not affect the VIM forms I and II suggests the existence of splice variants of vimentin.

Immunoprecipitation of VIM from TALH-STD and –NaCl cells using monoclonal anti-VIM antibody and protein G-Agarose matrix was performed. As expected the data showed protein band in the same molecular weight as VIM I–IV (Fig. S5 A). Using mass spectrometry and data bank search we could identify with highly significant scores the corresponding protein bands as vimentin. Moreover mass spectrometric sequencing of the VIM tryptic digest achieved 67.72% sequence coverage of VIM (Fig. S5 A). Furthermore using MALDI-TOF MS we generated peptide mass finger print form the tryptic digest of VIM III and IV and compared them to ones generated from the VIM I and II. The overlapping of the mass spectra clearly showed differences between the VIM forms suggesting peptide sequence heterogeneity between the VIM forms and supporting the existence of VIM splice variants (data Fig. S5 B, C).

### VIM Forms Play an Important Role in Apoptosis Induction and Resistance in Renal Cells

**Figure 6:**
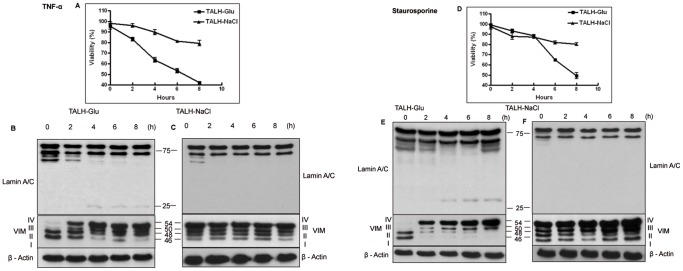
Expression of VIM and cell viability analysis of TALH cells during apoptosis induction. Western blot analysis of vimetin and lamin A/C expression in A: TALH-Glu and B: TALH-NaCl cells after 0, 2, 4, 6, and 8 h of treatment with 100 ng/ml TNF-α together with 10 µg/ml cycloheximide. C: Cell viability, determined with MTT assay, showed an increased survival in TALH-NaCl cells compared to TALH-STD and TALH-Glu cells in a time-dependent manner from 0 to 8 hr with TNF-α treatment. D: TALH-Glu and E: TALH-NaCl cells in the course of apoptosis induction with 1 µM staurosporine. F: Cell viability, determined with MTT assay, showed an increased resistance in TALH-NaCl cells compared to TALH-STD and TALH-Glu cells in a time-dependent manner from 0 to 8 hr with TNF-α treatment. VIM bands of higher molecular weight appeared after 2 h of treatment with TNF-α and staurosporine in TALH-Glu cells. In addition, lamin A/C was cleaved in a 28-kDa fragment (arrowhead) by caspase activation after 4 h. However, TNF-α and staurosporine treatments showed neither expression change in VIM forms nor cleavage products of lamin A/C in TALH-NaCl cells.

In order to understand the role of VIM forms in cell survival and adaptation, we induced apoptosis by the extrinsic pathway with TNF-α and the intrinsic (mitochondrial) pathway with staurosporine and monitored the expression of VIM and cell viability in a time-dependent manner for 0 to 8 h. Apoptosis induction was controlled with MTT cell viability assay and the caspase 6-determined cleavage fragments of lamin A/C [25]. The cell viability analysis showed that TALH-Glu cells were more prone to death, with a decrease in cell viability from 100% to 48% under TNF-α treatment (Fig. 6A) and to 44% under staurosporine (Fig. 6D). In contrast, TALH-NaCl cells were more resistant to apoptosis induction, from 100% to 79% under TNF-α treatment (Fig. 6A) and to 80% under staurosporine treatment (Fig. 6D). In parallel, western blot analysis of lamin A/C demonstrated that a cleavage product of 25 kDa in molecular weight appeared after 4 h of apoptosis induction in TALH-Glu (Fig. 6B, 6E) and after 6 h in TALH-STD (Fig. S6) cells but not in TALH-NaCl cells (Fig. 6C, 6F). Furthermore, we found an up-regulation in the high-molecular-weight forms of VIM in TALH-Glu cells compared to almost no difference in TALH-NaCl cells after 2 h of apoptosis induction; however, the lower-molecular-weight forms were down-regulated, illustrating the role of VIM form levels in renal cell resistance to apoptosis.

**Figure 7:**
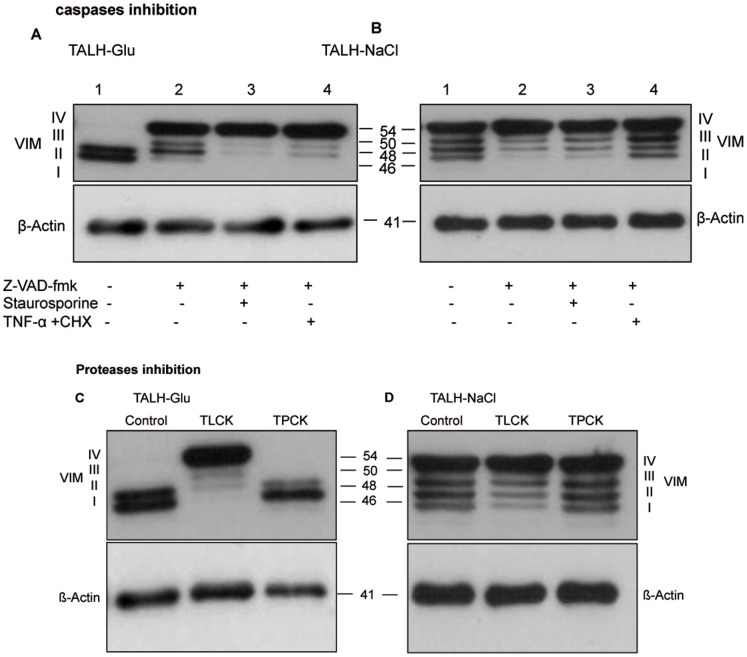
Western blot analysis of VIM after inhibition of caspases or proteases in TALH cells. A: TALH-cells adapted to high glucose (lane 1: control) were treated for 2 h with the pan-caspase inhibitor z-VAD-fmk (lane 2) and then 4 h with 1 µM staurosporine (lane 3) or 100 ng/ml TNF-α plus 10 µg/ml CHX (lane 4). B: TALH-cells adapted to high NaCl (lane 1: control) were treated for 2 h with the pan-caspase inhibitor z-VAD-fmk (lane 2) and then 4 h with 1 µM staurosporine (lane 3) or 100 ng/ml TNF-α plus 10 µg/ml CHX (lane 4). TALH cells (lane 1: control) were treated for 4 h with TLCK (lane 2) or TPCK (lane 3). C: TLCK but not TPCK influences the expression of VIM forms in TALH-Glu cells. D: TALH- NaCl showed no change in the expression of VIM forms. β-actin antibody was kept as control. The molecular mass in kDa is indicated at the left of each panel.

Caspases are the key effector proteins of the physiological death process, known as apoptosis. They exist in most of our cells as inactive precursors that lead to cell death once activated. Z-VAD-fmk is a pan-caspase inhibitor, which blocks apoptotic cell death. Treatment of cells with Z-VAD-fmk results in VIM expression alteration in TALH-Glu (Fig. 7A) cells but not in –NaCl once (Fig. 7B). This protein expression alteration was independent of apoptosis inducers. This supports the assumption that renal cells under glucose stress express low amounts of higher-molecular-weight forms of VIM and exhibit higher apoptosis levels, whereas renal cells that are exposed to higher NaCl concentrations express large amounts of higher-molecular-weight VIM and exhibit higher resistance to apoptosis. TLCK (Nα-Tosyl-Lys-chloromethylketone) and TPCK (N-Tosyl-L-phenylalanine chloromethyl) are active site-directed agents that irreversibly inhibit serine proteases. TALH-Glu cells that were treated with TLCK or TPCK showed an alteration of VIM expression under TLCK but not under TPCK treatment (Fig. 7C), in contrast the inhibitors treatments had no effect on VIM expression in TALH-NaCl cells (Fig. 7D).

## Discussion

The changes in osmolarity occur physiologically due to fluctuations in NaCl and urea concentrations and pathophysiologically due to glucosuria in patients suffering from diabetes mellitus [26]. As an important part in the urinary concentration mechanism, renal tubular cells are able to restore their cell volume to normal despite high changes in extracellular osmolarity [27]. However; their adaptive mechanism has remained poorly characterized, and the underlying signaling pathways are poorly explored. The present study provides further evidence for the involvement of cytoskeletal proteins, such as VIM, CFL, and CK, in renal cell resistance to osmotic stress conditions with altered NaCl and glucose concentrations.

Among these cytoskeletal proteins, the IF protein VIM showed expression alteration, accompanied by changes in localization, under NaCl and glucose stress conditions. The increase in VIM expression in NaCl-stressed cells seems to play an important role in the stabilization of the cell shape, as VIM is known to provide structural stability [28]. VIM is known to have the tendency to associate with other cytoskeletal components, membranes, and specific proteins [29], [30]. VIM can also interact directly with actin filaments [31]. It is already known that one of the aspects of osmotic stress resistance is to trigger cytoskeletal reorganization in the form of actin filament rearrangements [29], [30]. Furthermore, the regulation of CFL further strengthens the theory, as CFL has been shown to be an essential actin regulatory protein that severs filaments and accelerates actin assembly dynamics [32], [33].

Apart from the role of VIM in terms of interaction with actin filaments, VIM is also known to affect the localization and activity of the sodium-glucose cotransporter SGLT 1 [34] and is thus involved in glucose metabolism in renal proximal tubular cells. The strong regulation of the 57-kDa protein VIM in osmotic stress adaptation has been described in the form of augmented synthesis or assembly of soluble subunits of VIM by Soellner et al. [35]. Further evidence for the involvement of VIM in osmotic stress adaptation was provided by studies with VIM-deficient mice, in which altered taurine release after hypotonic stress could be observed [36].

IFs are known to take part in the distribution of caspases, as the effector proteases in apoptosis [37] and VIM form a complex with the proapoptotic p53, whose cleavage leads to nuclear translocation of p53 and apoptosis induction [38]. Increased resistance of TALH-NaCl cells to apoptotic induction with TNF-α compared to TALH-Glu cells and TALH-STD cells gave evidence for the reverse involvement of VIM forms in apoptosis. This is evidenced by the overexpression of the higher-molecular-weight VIM forms (VIM III and VIM IV) in TALH-NaCl cells compared to control and TALH-Glu cells. The independent regulation of the vimentin forms under stress conditions is in contrast to previous data that showed that VIM is prone to be cleaved in response to certain stress stimuli, producing different molecular weight forms of VIM as apoptotic products [39]. As has been described before, hyperosmotic stress can stimulate TNF-α expression [40], which could be the possible reason for the higher viability of TALH-NaCl cells that are used to higher levels of TNF-α. Higher viability of TALH-NaCl cells was also observed with staurosporine treatment, which causes apoptosis independently of TNF-α. Moreover, the reappearance of these higher-molecular-weight VIM forms in TALH-Glu cells during the course of apoptotic induction further showed that cells started a self defence mechanism against apoptotic events by increasing the expression of higher-molecular-weight forms of VIM.

Glucose is also known to induce reactive oxygen species via protein kinase C (PKC) activation [41], which makes cells more vulnerable to other stresses. VIM is known to be phosphorylated by PKC, which is accompanied by the reorganization of intracellular membranes [42]. Our results for protease inhibition indicate an involvement of PKC in osmotic stress adaptation, as TLCK, a potent inhibitor of PKC, had an impact on cleavage forms of VIM but not TPCK [43]. As VIM is regulated in osmotic stress as well as in apoptosis induction and protease inhibition in TALH-Glu cells, it seems likely that multiple proteases systems that influence VIM are altered in TALH cells that are adapted to high glucose.

Taken together, our study demonstrates that different forms of VIM are more than only apoptotic products in different kidney cell lines, as they are present in standard conditions of osmolarity. Moreover, VIM and the level of its different forms are closely related to cell resistance to osmotic stress and apoptosis and play an important role in the osmotic stress adaptation of renal cells.

## Supporting Information

Figure S1 **2-DE protein map of total proteins isolated from TALH-Glu cells.** 150 µg protein was loaded on a 11-cm IPG strip with a linear pH 5–8 gradient for isoelectric focusing, and a 12% SDS polyacrylamide gel was used for SDS-PAGE. Proteins were stained with flamingo. Selected proteins that were found to be differently expressed in TALH-Glu cells compared to TALH-STD cells, were assigned a number corresponding to their number in Table 1.
(TIF)

Figure S2 **Distribution of VIM in TALH-Cells.** A: Immunofluorescence staining of TALH-cells using mouse anti-VIM antibody in TALH-STD, TALH-NaCl, TALH-Glucose cells. Images were performed using confocal microscope FV1000 from Olympus (Olympus Optical, Hamburg Germany) Mikroskop FV1000 von Olympus (Olympus Optical, Hamburg, Deutschland). The images were carried out using 60x objective. Red: vimentin and blue: DAPI nucleus staining. The image reconstruction was carried out using Imaris x64, Version 7.4.0 (Bitplane, Zurich, Switzerland). B: Immunofluorescence staining of TALH-cells using mouse anti-VIM (a, b, c, d), anti-CFL (e, f, g, h), and anti-CK (i, j, k, l) antibodies in TALH-STD, TALH-NaCl, TALH-Glucose, and TALH-Urea cells, respectively. VIM builds a strong filamentous network in TALH-NaCl (b) cells compared to strong perinuclear restriction in TALH-Glu (c) cells. Scale bar, 20 µm. Images were performed using inverted immunofluorescence Zeiss Axiophot microscope (Carl Zeiss, Jena, Germany) equipped for epifluorescence with objectives ranging from magnifications of 10× to 100× with oil-immersion and a black and white Zeiss Axiocam CCD camera. Image capture was carried out using AnalySIS software (Soft Imaging Systems, Leinfelden, Germany).
(TIF)

Figure S3 **Expression analysis of VIM under stress conditions.** A: 2D Western blot analysis of vimentin expression in TALH-STD cells in the course of hyperosmolar NaCl stress. TALH-STD cells were stressed with 600 mosmol/kg NaCl medium and tested for vimentin expression after 0, 24, 48, 72 and 96 h. Acidic forms of vimentin are regulated during hyperosmolar NaCl stress. B: Immunofluorescence staining of VIM in TALH cells after 72 h of hypoosmotic stress. Images were performed using confocal microscope FV1000 from Olympus (Olympus Optical, Hamburg Germany) Mikroskop FV1000 von Olympus (Olympus Optical, Hamburg, Deutschland). The images were carried out using 60x objective. Red: vimentin and blue: DAPI nucleus staining. The image reconstruction was carried out using Imaris x64, Version 7.4.0 (Bitplane, Zurich, Switzerland). Arrows indicate colocalisation of VIM with membrane and the VIM in nucleus.
(TIF)

Figure S4 **VIM knock-down using siRNA.** A: VIM mRNA sequence with the biding positions of three used siRNAs. B: Western blot analysis of VIM in non-transfected (Ctr) and TALH cells transfected with the VIM siRNAs 1, 2, 3 or all three combined. C: The monitoring of apoptosis in siRNA TALH-cells subjected to osmotic stress was carried out using Western blot for caspase 8 and 3.
(TIF)

Figure S5 **Immunoprecipitation and MS analysis of VIM forms.** A: left panel, Immunoprecipitation of VIM from TALH-STD and –NaCl cells using monoclonal anti-VIM antibody and protein G-Agarose matrix. SDS-PAGE from immunoprecipitated proteins showed the four different forms of VIM. Right panel, Mass spectrometric sequencing of the VIM tryptic digest achieved 67.72% sequence coverage of VIM. B, C: MALDI-TOF MS analyses of the tryptic digest from VIM I, II, III and IV. The mass spectra from the different forms were generated and overlapped to illustrate the differences between the VIM forms. An Applied Biosystems Voyager-DE STR time-of-flight mass spectrometer, operating in delayed reflector mode with an accelerated voltage of 20 kV, was used to generate peptide mass fingerprints.
(TIF)

Figure S6 **Impact of apoptosis on VIM expression.** Western blot analysis of vimentin in TALH-STD cells during apoptosis induction. A: TALH-STD cells were probed with vimentin or lamin A/C antibody after 0, 2, 4, 6 and 8 h treatment with 100 ng/ml TNF-α and 10 µg/ml cycloheximide (CHX). B: TALH-STD cells were probed with vimentin or lamin A/C antibody after 0, 2, 4, 6 and 8 h treatment with 1 µM staurosporine. lamin A/C is cleaved in a 28 kDa fragment (arrowhead) by caspase activation after 4 h.
(TIF)

## References

[1] Cornet M, Lambert IH, Hoffmann EK (1993) Relation between cytoskeleton, hypo-osmotic treatment and volume regulation in Ehrlich ascites tumor cells. J Membr Biol 131: 55–66. 8433352 10.1007/BF02258534

[2] Krapivinsky GB, Ackerman MJ, Gordon EA, Krapivinsky LD, Clapham DE (1994) Molecular characterization of a swelling-induced chloride conductance regulatory protein, pICln. Cell 76: 439–448. 8313467 10.1016/0092-8674(94)90109-0

[3] Bibi A, Agarwal NK, Dihazi GH, Eltoweissy M, Van Nguyen P, et al. Calreticulin is crucial for calcium homeostasis mediated adaptation and survival of thick ascending limb of Henle’s loop cells under osmotic stress. Int J Biochem Cell Biol 43: 1187–1197. 10.1016/j.biocel.2011.04.012 21554974

[4] Wehner F, Olsen H, Tinel H, Kinne-Saffran E, Kinne RK (2003) Cell volume regulation: osmolytes, osmolyte transport, and signal transduction. Rev Physiol Biochem Pharmacol 148: 1–80. 12687402 10.1007/s10254-003-0009-x

[5] Garcia-Perez A, Burg MB (1991) Renal medullary organic osmolytes. Physiol Rev 71: 1081–1115. 1924548 10.1152/physrev.1991.71.4.1081

[6] Bustamante M, Roger F, Bochaton-Piallat ML, Gabbiani G, Martin PY, et al (2003) Regulatory volume increase is associated with p38 kinase-dependent actin cytoskeleton remodeling in rat kidney MTAL. Am J Physiol Renal Physiol 285: F336–347. 12724128 10.1152/ajprenal.00003.2003

[7] Di Ciano C, Nie Z, Szaszi K, Lewis A, Uruno T, et al (2002) Osmotic stress-induced remodeling of the cortical cytoskeleton. Am J Physiol Cell Physiol 283: C850–865. 12176742 10.1152/ajpcell.00018.2002

[8] Dihazi H, Asif AR, Agarwal NK, Doncheva Y, Muller GA (2005) Proteomic analysis of cellular response to osmotic stress in thick ascending limb of Henle’s loop (TALH) cells. Mol Cell Proteomics 4: 1445–1458. 15975915 10.1074/mcp.M400184-MCP200

[9] Janmey PA (1998) The cytoskeleton and cell signaling: component localization and mechanical coupling. Physiol Rev 78: 763–781. 9674694 10.1152/physrev.1998.78.3.763

[10] Skalli O, Goldman RD (1991) Recent insights into the assembly, dynamics, and function of intermediate filament networks. Cell Motil Cytoskeleton 19: 67–79. 1878980 10.1002/cm.970190202

[11] Sarria AJ, Nordeen SK, Evans RM (1990) Regulated expression of vimentin cDNA in cells in the presence and absence of a preexisting vimentin filament network. J Cell Biol 111: 553–565. 1696263 10.1083/jcb.111.2.553 PMC2116208

[12] Albers K, Fuchs E (1987) The expression of mutant epidermal keratin cDNAs transfected in simple epithelial and squamous cell carcinoma lines. J Cell Biol 105: 791–806. 2442174 10.1083/jcb.105.2.791 PMC2114764

[13] Ando J, Sugimoto K, Tamayose K, Sasaki M, Ando M, et al (2008) Changes in cell morphology and cytoskeletal organization are induced by human mitotic checkpoint gene, Bub1. Biochem Biophys Res Commun 365: 691–697. 18036341 10.1016/j.bbrc.2007.11.053

[14] Herrmann H, Fouquet B, Franke WW (1989) Expression of intermediate filament proteins during development of Xenopus laevis. I. cDNA clones encoding different forms of vimentin. Development 105: 279–298. 2806127 10.1242/dev.105.2.279

[15] Welch WJ, Feramisco JR, Blose SH (1985) The mammalian stress response and the cytoskeleton: alterations in intermediate filaments. Ann N Y Acad Sci 455: 57–67. 3866510 10.1111/j.1749-6632.1985.tb50403.x

[16] Ball EH, Singer SJ (1981) Association of microtubules and intermediate filaments in normal fibroblasts and its disruption upon transformation by a temperature-sensitive mutant of Rous sarcoma virus. Proc Natl Acad Sci U S A 78: 6986–6990. 6273900 10.1073/pnas.78.11.6986 PMC349178

[17] Eckes B, Colucci-Guyon E, Smola H, Nodder S, Babinet C, et al (2000) Impaired wound healing in embryonic and adult mice lacking vimentin. J Cell Sci 113 (Pt 13): 2455–2462. 10.1242/jcs.113.13.2455 10852824

[18] Ivaska J, Vuoriluoto K, Huovinen T, Izawa I, Inagaki M, et al (2005) PKCepsilon-mediated phosphorylation of vimentin controls integrin recycling and motility. Embo J 24: 3834–3845. 16270034 10.1038/sj.emboj.7600847 PMC1283946

[19] Kokkinos MI, Wafai R, Wong MK, Newgreen DF, Thompson EW, et al (2007) Vimentin and epithelial-mesenchymal transition in human breast cancer–observations in vitro and in vivo. Cells Tissues Organs 185: 191–203. 17587825 10.1159/000101320

[20] Scott DM (1987) Differentiation in vitro of primary cultures and transfected cell lines of epithelial cells derived from the thick ascending limb of Henle’s loop. Differentiation 36: 35–46. 2451630 10.1111/j.1432-0436.1987.tb00179.x

[21] Ryan MJ, Johnson G, Kirk J, Fuerstenberg SM, Zager RA, et al (1994) HK-2: an immortalized proximal tubule epithelial cell line from normal adult human kidney. Kidney Int 45: 48–57. 8127021 10.1038/ki.1994.6

[22] Muller GA, Frank J, Rodemann HP, Engler-Blum G (1995) Human renal fibroblast cell lines (tFKIF and tNKF) are new tools to investigate pathophysiologic mechanisms of renal interstitial fibrosis. Exp Nephrol 3: 127–133. 7773632

[23] Bradford MM (1976) A rapid and sensitive method for the quantitation of microgram quantities of protein utilizing the principle of protein-dye binding. Anal Biochem 72: 248–254. 942051 10.1016/0003-2697(76)90527-3

[24] Towbin H, Staehelin T, Gordon J (1979) Electrophoretic transfer of proteins from polyacrylamide gels to nitrocellulose sheets: procedure and some applications. Proc Natl Acad Sci U S A 76: 4350–4354. 388439 10.1073/pnas.76.9.4350 PMC411572

[25] Rao L, Perez D, White E (1996) Lamin proteolysis facilitates nuclear events during apoptosis. J Cell Biol 135: 1441–1455. 8978814 10.1083/jcb.135.6.1441 PMC2133948

[26] Bakris GL, Fonseca VA, Sharma K, Wright EM (2009) Renal sodium-glucose transport: role in diabetes mellitus and potential clinical implications. Kidney Int 75: 1272–1277. 19357717 10.1038/ki.2009.87

[27] Grunewald RW, Fahr M, Fiedler GM, Jehle PM, Muller GA (2001) Volume regulation of thick ascending limb of Henle cells: significance of organic osmolytes. Exp Nephrol 9: 81–89. 11150856 10.1159/000052598

[28] Eckes B, Dogic D, Colucci-Guyon E, Wang N, Maniotis A, et al (1998) Impaired mechanical stability, migration and contractile capacity in vimentin-deficient fibroblasts. J Cell Sci 111 (Pt 13): 1897–1907. 10.1242/jcs.111.13.1897 9625752

[29] Stamenkovic I, Skalli O, Gabbiani G (1986) Distribution of intermediate filament proteins in normal and diseased human glomeruli. Am J Pathol 125: 465–475. 2432791 PMC1888470

[30] Correia I, Chu D, Chou YH, Goldman RD, Matsudaira P (1999) Integrating the actin and vimentin cytoskeletons. adhesion-dependent formation of fimbrin-vimentin complexes in macrophages. J Cell Biol 146: 831–842. 10459017 10.1083/jcb.146.4.831 PMC2156141

[31] Esue O, Carson AA, Tseng Y, Wirtz D (2006) A direct interaction between actin and vimentin filaments mediated by the tail domain of vimentin. J Biol Chem 281: 30393–30399. 16901892 10.1074/jbc.M605452200

[32] Michelotti A, Farella M, Buonocore G, Pellegrino G, Piergentili C, et al (2007) Is unilateral posterior crossbite associated with leg length inequality? Eur J Orthod 29: 622–626. 17873143 10.1093/ejo/cjm071

[33] Roland J, Berro J, Michelot A, Blanchoin L, Martiel JL (2008) Stochastic severing of actin filaments by actin depolymerizing factor/cofilin controls the emergence of a steady dynamical regime. Biophys J 94: 2082–2094. 18065447 10.1529/biophysj.107.121988 PMC2257902

[34] Runembert I, Queffeulou G, Federici P, Vrtovsnik F, Colucci-Guyon E, et al (2002) Vimentin affects localization and activity of sodium-glucose cotransporter SGLT1 in membrane rafts. J Cell Sci 115: 713–724. 11865027 10.1242/jcs.115.4.713

[35] Soellner P, Quinlan RA, Franke WW (1985) Identification of a distinct soluble subunit of an intermediate filament protein: tetrameric vimentin from living cells. Proc Natl Acad Sci U S A 82: 7929–7933. 3865206 10.1073/pnas.82.23.7929 PMC390883

[36] Ding M, Eliasson C, Betsholtz C, Hamberger A, Pekny M (1998) Altered taurine release following hypotonic stress in astrocytes from mice deficient for GFAP and vimentin. Brain Res Mol Brain Res 62: 77–81. 9795147 10.1016/s0169-328x(98)00240-x

[37] Dinsdale D, Lee JC, Dewson G, Cohen GM, Peter ME (2004) Intermediate filaments control the intracellular distribution of caspases during apoptosis. Am J Pathol 164: 395–407. 14742246 10.1016/S0002-9440(10)63130-6 PMC1602261

[38] Yang X, Wang J, Liu C, Grizzle WE, Yu S, et al (2005) Cleavage of p53-vimentin complex enhances tumor necrosis factor-related apoptosis-inducing ligand-mediated apoptosis of rheumatoid arthritis synovial fibroblasts. Am J Pathol 167: 705–719. 16127151 10.1016/S0002-9440(10)62045-7 PMC1698724

[39] Byun Y, Chen F, Chang R, Trivedi M, Green KJ, et al (2001) Caspase cleavage of vimentin disrupts intermediate filaments and promotes apoptosis. Cell Death Differ 8: 443–450. 11423904 10.1038/sj.cdd.4400840

[40] Kelly DJ, Aaltonen P, Cox AJ, Rumble JR, Langham R, et al (2002) Expression of the slit-diaphragm protein, nephrin, in experimental diabetic nephropathy: differing effects of anti-proteinuric therapies. Nephrol Dial Transplant 17: 1327–1332. 12105259 10.1093/ndt/17.7.1327

[41] Xia L, Wang H, Munk S, Kwan J, Goldberg HJ, et al (2008) High glucose activates PKC-zeta and NADPH oxidase through autocrine TGF-beta1 signaling in mesangial cells. Am J Physiol Renal Physiol 295: F1705–1714. 18815221 10.1152/ajprenal.00043.2008

[42] Takai Y, Ogawara M, Tomono Y, Moritoh C, Imajoh-Ohmi S, et al (1996) Mitosis-specific phosphorylation of vimentin by protein kinase C coupled with reorganization of intracellular membranes. J Cell Biol 133: 141–149. 8601602 10.1083/jcb.133.1.141 PMC2120783

[43] Solomon DH, O’Brian CA, Weinstein IB (1985) N-alpha-Tosyl-L-lysine chloromethyl ketone and N-alpha-tosyl-L-phenylalanine chloromethyl ketone inhibit protein kinase C. FEBS Lett. 190: 342–344. 10.1016/0014-5793(85)81315-6 4043411

