# Correction: Renal Cells Express Different Forms of Vimentin: The Independent Expression Alteration of these Forms is Important in Cell Resistance to Osmotic Stress and Apoptosis

**DOI:** 10.1371/journal.pone.0196935

**Published:** 2018-05-01

**Authors:** Bettina S. Buchmaier, Asima Bibi, Gerhard A. Müller, Gry H. Dihazi, Marwa Eltoweissy, Jenny Kruegel, Hassan Dihazi

There is an affiliation missing for the fifth author. Marwa Eltoweissy is also affiliated with: Department of Zoology, Faculty of Science, Alexandria University, Alexandria, Egypt.
