# Supplementary figures and images for: Renal Cells Express Different Forms of Vimentin: The Independent Expression Alteration of these Forms is Important in Cell Resistance to Osmotic Stress and Apoptosis
Source: PLoS One. 2013 Jul 11;8(7):e68301. doi: 10.1371/journal.pone.0068301 (PMC3708942; doi:10.1371/journal.pone.0068301)

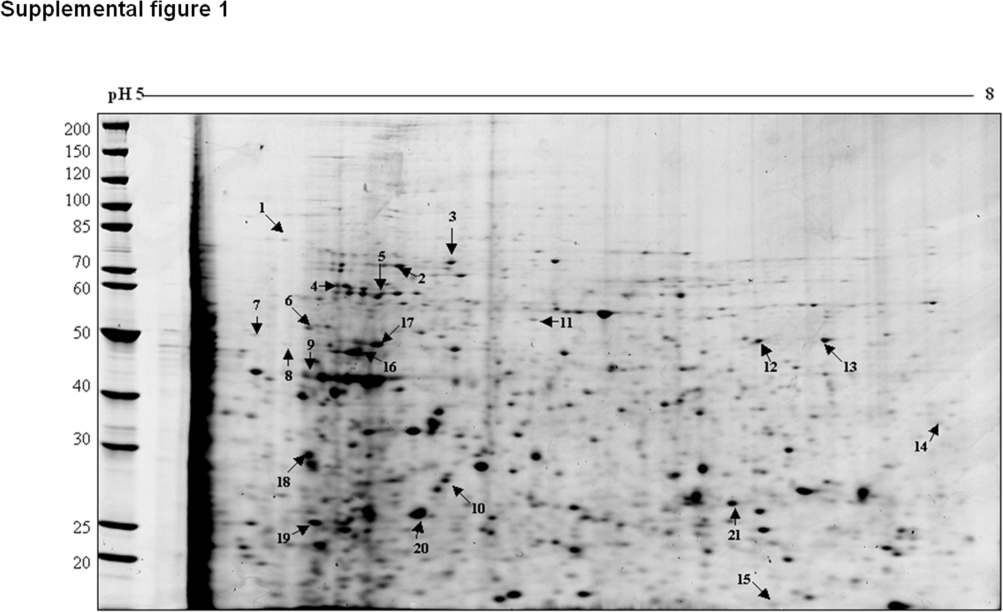

Supplement: Figure S1 — 2-DE protein map of total proteins isolated from TALH-Glu cells. 150 µg protein was loaded on a 11-cm IPG strip with a linear pH 5–8 gradient for isoelectric focusing, and a 12% SDS polyacrylamide gel was used for SDS-PAGE. Proteins were stained with flamingo. Selected proteins that were found to be differently expressed in TALH-Glu cells compared to TALH-STD cells, were assigned a number corresponding to their number in Table 1. (TIF) [file pone.0068301.s001.tif]

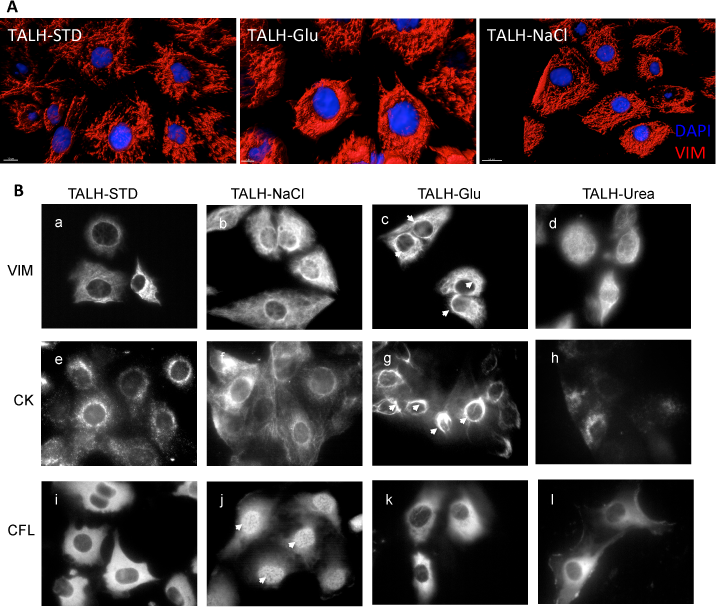

Supplement: Figure S2 — Distribution of VIM in TALH-Cells. A: Immunofluorescence staining of TALH-cells using mouse anti-VIM antibody in TALH-STD, TALH-NaCl, TALH-Glucose cells. Images were performed using confocal microscope FV1000 from Olympus (Olympus Optical, Hamburg Germany) Mikroskop FV1000 von Olympus (Olympus Optical, Hamburg, Deutschland). The images were carried out using 60x objective. Red: vimentin and blue: DAPI nucleus staining. The image reconstruction was carried out using Imaris x64, Version 7.4.0 (Bitplane, Zurich, Switzerland). B: Immunofluorescence staining of TALH-cells using mouse anti-VIM (a, b, c, d), anti-CFL (e, f, g, h), and anti-CK (i, j, k, l) antibodies in TALH-STD, TALH-NaCl, TALH-Glucose, and TALH-Urea cells, respectively. VIM builds a strong filamentous network in TALH-NaCl (b) cells compared to strong perinuclear restriction in TALH-Glu (c) cells. Scale bar, 20 µm. Images were performed using inverted immunofluorescence Zeiss Axiophot microscope (Carl Zeiss, Jena, Germany) equipped for epifluorescence with objectives ranging from magnifications of 10× to 100× with oil-immersion and a black and white Zeiss Axiocam CCD camera. Image capture was carried out using AnalySIS software (Soft Imaging Systems, Leinfelden, Germany). (TIF) [file pone.0068301.s002.tif]

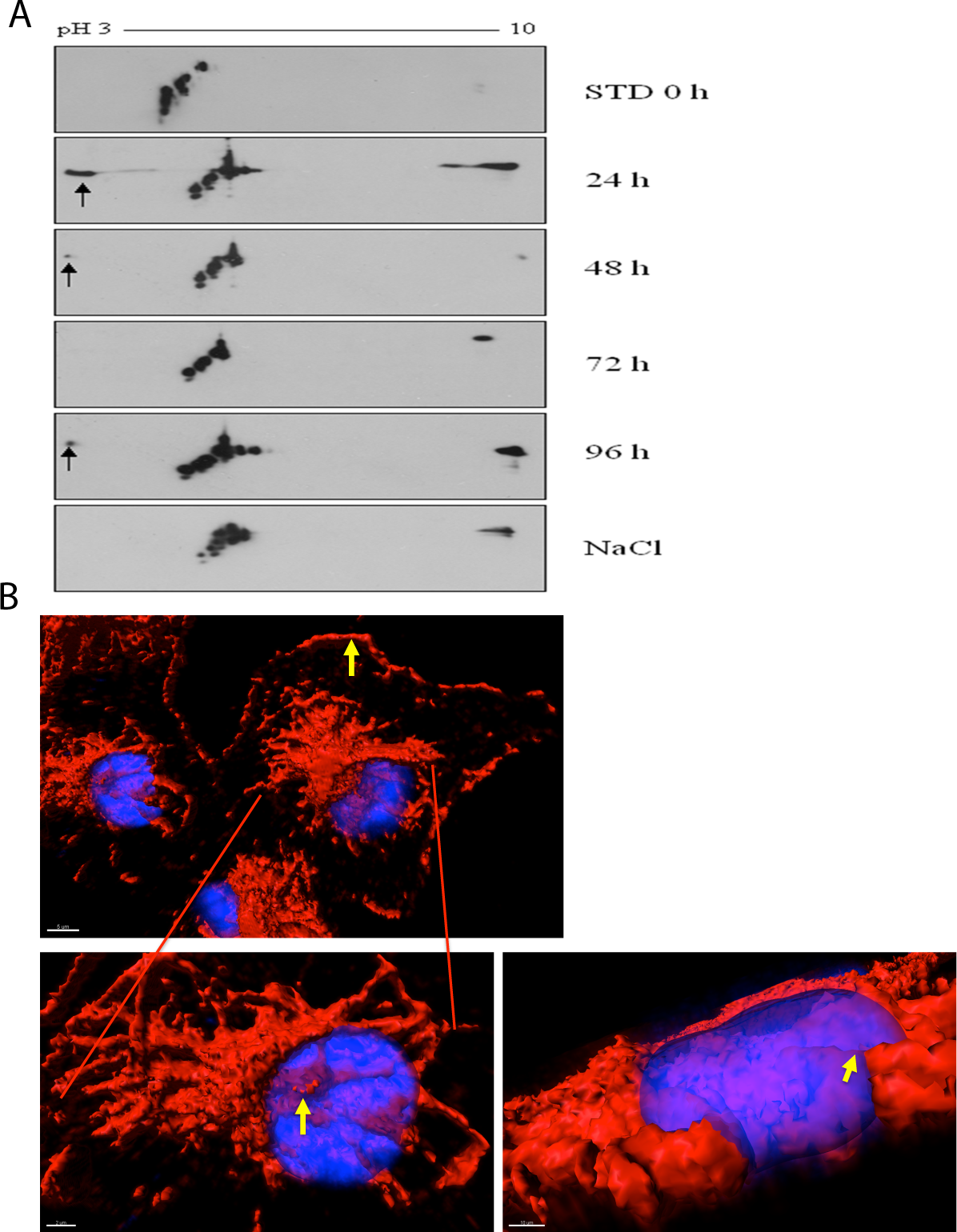

Supplement: Figure S3 — Expression analysis of VIM under stress conditions. A: 2D Western blot analysis of vimentin expression in TALH-STD cells in the course of hyperosmolar NaCl stress. TALH-STD cells were stressed with 600 mosmol/kg NaCl medium and tested for vimentin expression after 0, 24, 48, 72 and 96 h. Acidic forms of vimentin are regulated during hyperosmolar NaCl stress. B: Immunofluorescence staining of VIM in TALH cells after 72 h of hypoosmotic stress. Images were performed using confocal microscope FV1000 from Olympus (Olympus Optical, Hamburg Germany) Mikroskop FV1000 von Olympus (Olympus Optical, Hamburg, Deutschland). The images were carried out using 60x objective. Red: vimentin and blue: DAPI nucleus staining. The image reconstruction was carried out using Imaris x64, Version 7.4.0 (Bitplane, Zurich, Switzerland). Arrows indicate colocalisation of VIM with membrane and the VIM in nucleus. (TIF) [file pone.0068301.s003.tif]

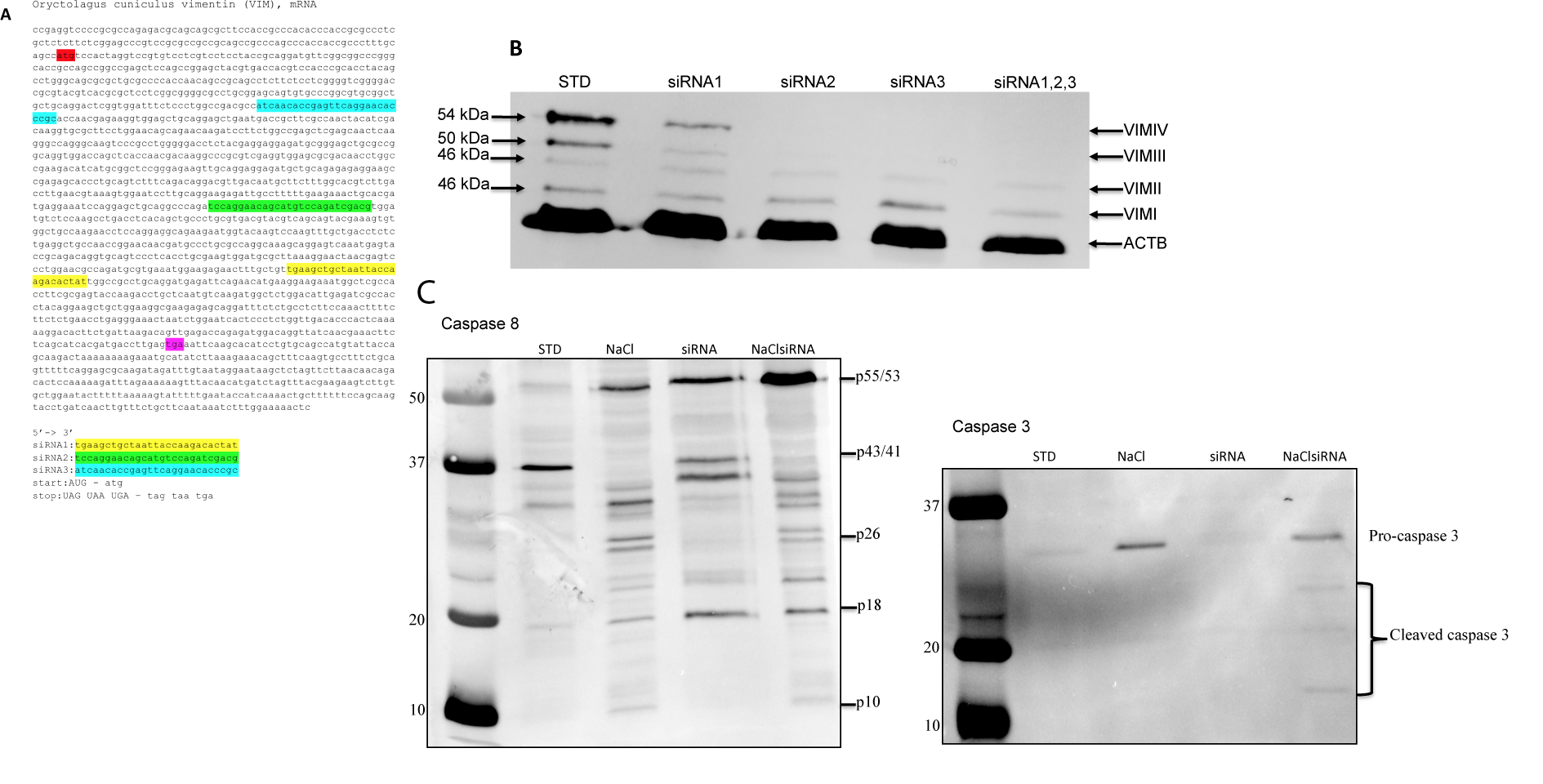

Supplement: Figure S4 — VIM knock-down using siRNA. A: VIM mRNA sequence with the biding positions of three used siRNAs. B: Western blot analysis of VIM in non-transfected (Ctr) and TALH cells transfected with the VIM siRNAs 1, 2, 3 or all three combined. C: The monitoring of apoptosis in siRNA TALH-cells subjected to osmotic stress was carried out using Western blot for caspase 8 and 3. (TIF) [file pone.0068301.s004.tif]

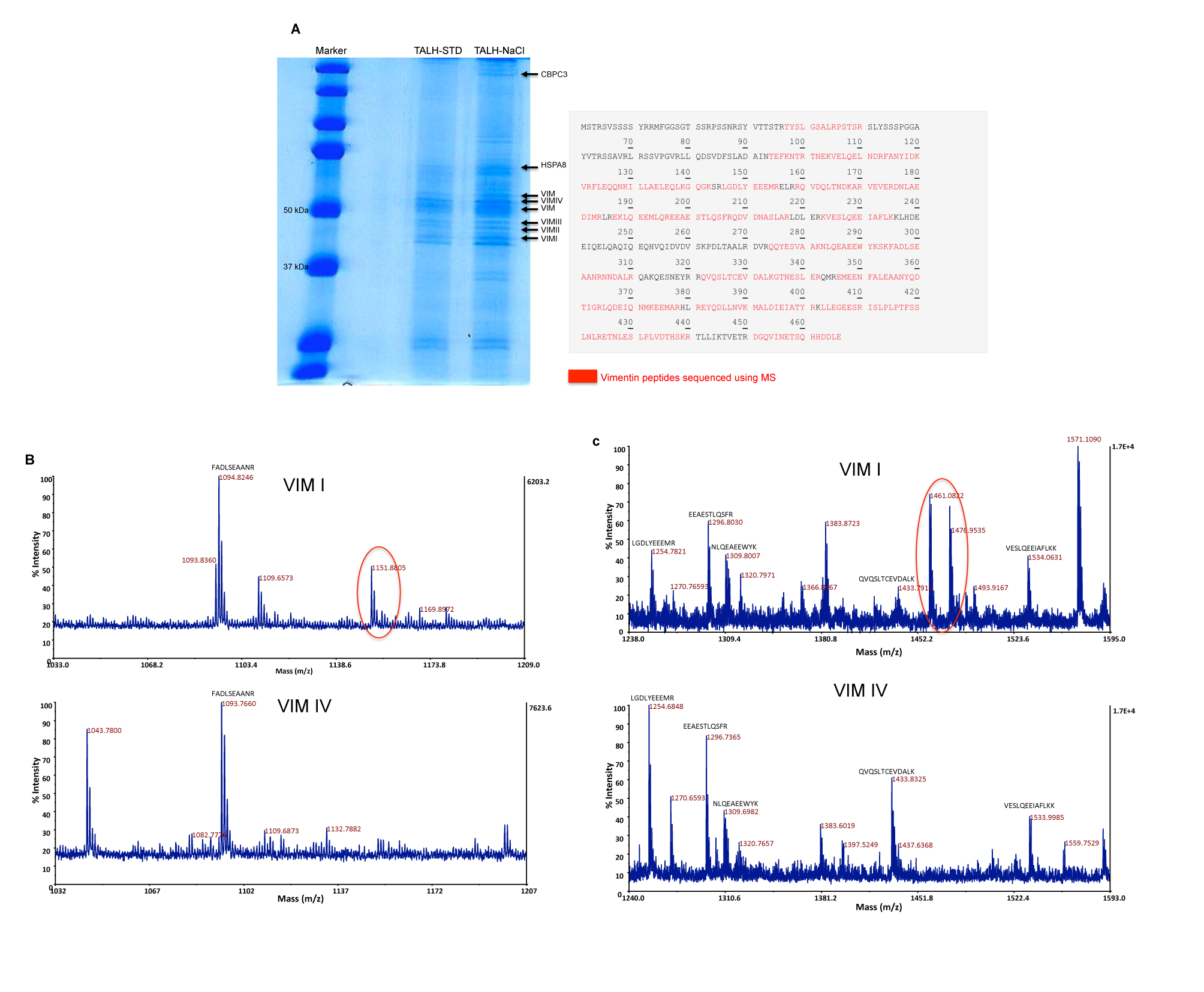

Supplement: Figure S5 — Immunoprecipitation and MS analysis of VIM forms. A: left panel, Immunoprecipitation of VIM from TALH-STD and –NaCl cells using monoclonal anti-VIM antibody and protein G-Agarose matrix. SDS-PAGE from immunoprecipitated proteins showed the four different forms of VIM. Right panel, Mass spectrometric sequencing of the VIM tryptic digest achieved 67.72% sequence coverage of VIM. B, C: MALDI-TOF MS analyses of the tryptic digest from VIM I, II, III and IV. The mass spectra from the different forms were generated and overlapped to illustrate the differences between the VIM forms. An Applied Biosystems Voyager-DE STR time-of-flight mass spectrometer, operating in delayed reflector mode with an accelerated voltage of 20 kV, was used to generate peptide mass fingerprints. (TIF) [file pone.0068301.s005.tif]

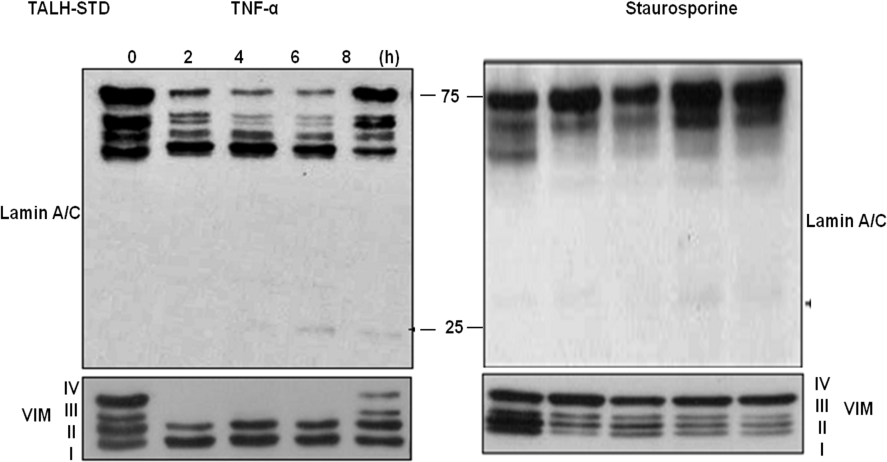

Supplement: Figure S6 — Impact of apoptosis on VIM expression. Western blot analysis of vimentin in TALH-STD cells during apoptosis induction. A: TALH-STD cells were probed with vimentin or lamin A/C antibody after 0, 2, 4, 6 and 8 h treatment with 100 ng/ml TNF-α and 10 µg/ml cycloheximide (CHX). B: TALH-STD cells were probed with vimentin or lamin A/C antibody after 0, 2, 4, 6 and 8 h treatment with 1 µM staurosporine. lamin A/C is cleaved in a 28 kDa fragment (arrowhead) by caspase activation after 4 h. (TIF) [file pone.0068301.s006.tif]
